# Evaluation of an effectiveness and safety of the electroacupuncture in the management of intractable neuropathic pain: A prospective, open-labeled, randomized, cross-over clinical trial

**DOI:** 10.1097/MD.0000000000023725

**Published:** 2020-12-18

**Authors:** Jee Youn Moon, Chang-Soon Lee, Yongjae Yoo, Suji Lee, Sang Hoon Lee, Seunghoon Lee

**Affiliations:** a Department of Anesthesiology and Pain Medicine, Seoul National University Hospital College of Medicine; b Department of Clinical Korean Medicine, Graduate School, Kyung Hee University; c Department of Acupuncture and Moxibustion Medicine, Kyung Hee University Korean Medicine Hospital; d Department of Acupuncture and Moxibustion, College of Korean Medicine, Kyung Hee University, Seoul, Republic of Korea.

**Keywords:** electroacupuncture, neuropathic pain, randomized clinical trial, study protocol

## Abstract

**Background:**

There is no sufficient evidence on the effectiveness of acupuncture for neuropathic pain. This protocol describes a study that aims to evaluate the effectiveness and safety of electroacupuncture combined with conventional medicine for patients with intractable neuropathic pain, when compared with conventional medicine alone.

**Methods/design:**

This study is a prospective, open-labeled, randomized, cross-over clinical trial. A total of 40 patients with neuropathic pain who had a numeric rating scale (NRS) score of over 4 despite receiving conventional treatment for more than 3 months will be enrolled. Participants will receive conventional treatment for neuropathic pain (treatment C) or treatment C combined with 12 additional sessions of electroacupuncture treatment (treatment A) for 6 weeks. Participants will be randomly assigned to 1 of the 2 sequence groups (AC and CA group) with a 1:1 allocation. The differences of responder in the composite efficacy outcomes, which consist of the NRS, Brief Pain Inventory-Short Form (BPI-SF) pain subscale, and global assessment at 6 weeks after randomization will be examined as the primary outcome. Secondary outcomes include differences in the NRS, the Short-Form McGill Pain Questionnaire, BPI-SF, Fatigue Severity Scale, Hospital Anxiety and Depression Scale, Medical Outcomes Study Sleep Scale, global assessment, EQ-5D, and incremental cost-effective ratio at 6 and 15 weeks after randomization. Adverse events, vital signs, and physical examinations will be recorded to evaluate safety.

**Discussion:**

The study protocol for this trial will provide up-to-date evidence on the effectiveness and safety of electroacupuncture for patients with intractable neuropathic pain. The results will be disseminated through a peer-reviewed journal and conference presentations.

**Trial registration:**

Clinical Research Information Service, ID: KCT0003615. Registered on March 12, 2019. https://cris.nih.go.kr/cris/search/search_result_st01_kren.jsp?seq=13410& ltype=&rtype=

## 1 Introduction

Neuropathic pain develops after injury to the nervous system, and is often accompanied by maladaptive changes in the somatosensory nervous system.^[1]^ The symptoms of neuropathic pain are often abnormally exaggerated by painful stimuli, and can even be provoked by non-painful stimuli. This could easily progress to chronic and refractory pain, which presents a great challenge in terms of management. Conventional pharmacological treatments for neuropathic pain include antidepressants and antiepileptics;^[2]^ however, the number of patients who achieve satisfactory pain relief is low, and is only 10% to 25% more than patients receiving placebo.^[3]^ Moreover, high comorbidity rates accompanied by chronic neuropathic pain, such as insomnia, anxiety, or depression,^[4]^ further makes the treatment challenging, and patients seek alternative treatment strategies including acupuncture treatment.^[5]^

Some studies have proposed the mechanisms of acupuncture for pain reduction. The local analgesic effects of acupuncture are mediated by adenosine A1 receptors^[6]^ or inactivation of the myofascial trigger point.^[7]^ Acupuncture analgesia can also be induced segmentally by the gate-control theory of pain. Needling of some acupuncture points located at the extremities can generally reduce pain through descending inhibitory pain control by serotonin and noradrenaline.^[8,9]^ Recently, clinical evidence on acupuncture for chronic pain, which is similar to nociceptive pain, has been well-established through many systematic reviews^[10–12]^ and clinical guidelines.^[13,14]^ A meta-analysis assessing 20,827 individual patient data from 39 trials suggested that acupuncture is an effective treatment option for chronic pain, including nonspecific musculoskeletal pain, osteoarthritis, headache, or shoulder pain, and that its effects persist over time, with only a small decrease at 1 year.^[11]^ The guidelines published by the American College of Physicians recommend that acupuncture should initially be selected for patients with chronic low back pain.^[13]^ However, when focusing on neuropathic pain such as diabetic neuropathy, chemotherapy-induced peripheral neuropathy, postherpetic neuralgia, or failed back surgery syndrome, there is no sufficient evidence on the effectiveness of acupuncture.^[3]^

Our pilot study^[15]^ explored effectiveness of electroacupuncture (EA) for patients with moderate to severe neuropathic pain despite already receiving conventional oral medications, and assessed the feasibility of a large-scale trial. The results showed that EA significantly decreased the intensity of pain in neuropathic patients, especially burning sensations, electric shock-like pain, and mechanical hyperalgesia. Furthermore, the number of acupuncture sessions was a significant factor for favorable results of reducing pain intensity. Based on our previous trial, we designed a large-scale, prospective, open-labeled, randomized, cross-over clinical trial to evaluate the effectiveness and safety of 12 sessions of EA for the treatment of neuropathic pain.

## 2 Methods and design

### 2.1 Objective

The aim of this study is to determine the effectiveness and safety of EA treatment for neuropathic pain when added to conventional neuropathic pain pharmacotherapies compared with no EA treatment. The pain intensity, function, quality of life, global assessment, and adverse events will also be assessed.

### 2.2 Design and setting

This study is a prospective, open-labeled, randomized, cross-over clinical trial conducted at Seoul National University Hospital (SNUH) Pain Center and Kyung Hee University Korean Medicine Hospital (KHUKMH) in South Korea.

#### 2.2.1 Recruitment strategy

A total of 40 patients with chronic neuropathic pain who did not achieve satisfactory pain relief despite receiving conventional treatment for more than 3 months will be recruited in SNUH Pain Center. The expected recruitment period is from December 2019 to May 2021. Participants will be recruited through advertising on the bulletin boards of the hospital, internet homepages of the hospital, public institutions, and through the media.

#### 2.2.2 Study plan

This study is a cross-over trial that aims to compare conventional medical treatment (treatment C) with conventional medical treatment and an add-on EA treatment (treatment A) for patients with intractable neuropathic pain. A cross-over design was planned in which participants will be randomly assigned to 2 groups (AC and CA groups) according to the 2 treatment sequences.

**Figure 1:**
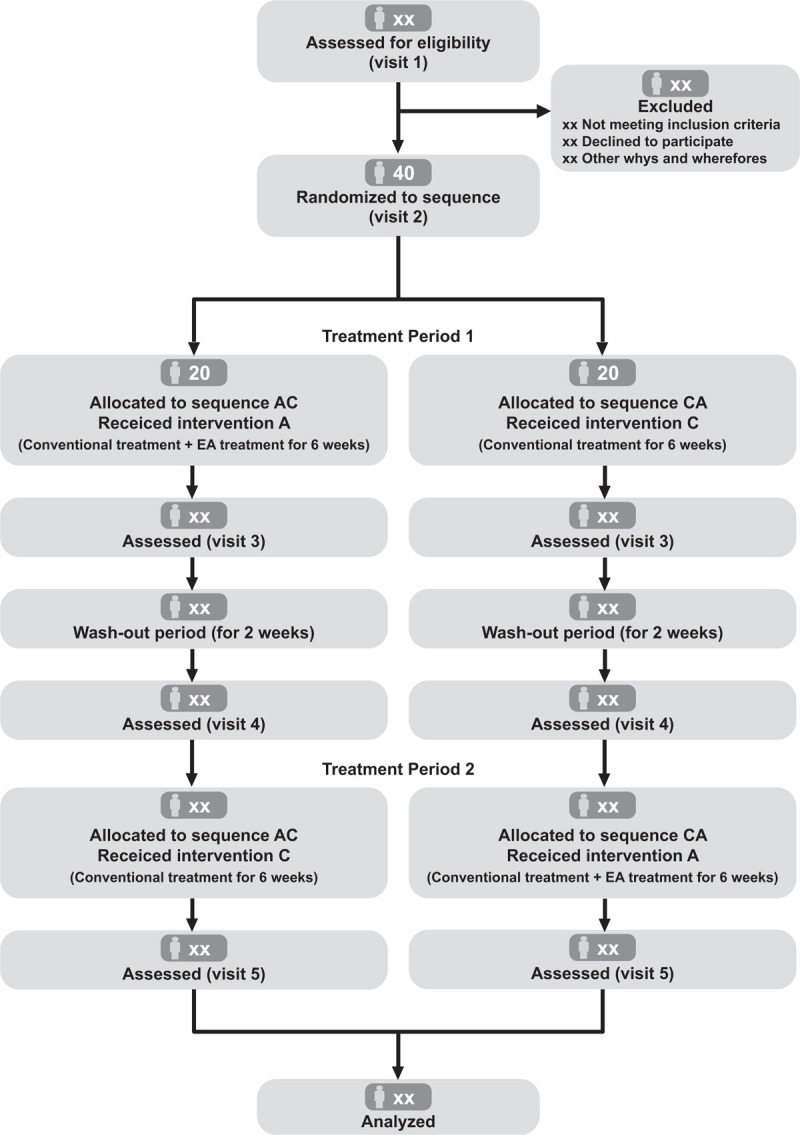
Modified CONSORT flow diagram for crossover trials. Intervention A: add-on electroacupuncture treatment; Intervention C; conventional treatment; EA: electroacupuncture.

Screening will be conducted after receiving written informed consent from the participant at SNUH Pain Center (Visit 1). They will then be randomly assigned to 1 of the 2 sequence groups: the AC group and CA group (Visit 2). The first treatment period will be conducted for 6 weeks. In the case of “treatment A”, during the treatment period, a total of 12 EA sessions will be performed for 6 weeks at KHUKMH. After the first treatment period, evaluations will continue at SNUH Pain Center (Visit 3). After a washout period of 2 weeks, participants will undergo a pain evaluation period for 7 days (Visit 4). If the average numeric rating scale (NRS) pain score, which is self-recorded in a diary, is 4 points or more during this period, patients will proceed to the second treatment period. If it is less than 4 points, the same “treatment C” will be maintained. The second treatment period will also be conducted for 6 weeks, as in the first treatment period, followed by a final evaluation (Visit 5). Figure 1 shows the study procedure and details.^[16]^

### 2.3 Types of participants

#### 2.3.1 Inclusion criteria

Participants who meet the following conditions will be included:

1. Patients aged 19 years and over at screening.
2. Patients who have peripheral neuropathic pain and satisfy criteria A) and B), and have criterion C) or D) identified on examination, depending on the conditions.
  A) Have a confirmed medical history of diagnosis for neuropathic pain according to clinical findings.
  B) Symptoms, history, and physical examination of patients suggest peripheral neuropathic pain.
  C) Electromyography/nerve conduction study or evoked potential test suggest peripheral neuropathy.
  D) Ultrasonographic examination at screening reveals lesions that indicate peripheral entrapment syndrome or peripheral neuropathy.
3. Have unsatisfactory pain relief despite appropriate conservative treatment for more than 3 months before screening.
4. Have a score ≥13 on the Korean version of the PainDETECT Questionnaire (PD-Q) at screening.
5. Have an average score ≥4 on the NRS measured by a pain diary during the 7-day screening period before randomization.
6. No evidence of disease recurrence in cancer survivors.
7. Patients who can voluntarily consent to the study, understand the process of the study, and adequately write a reportable questionnaire.

#### 2.3.2 Exclusion criteria

Participants will be excluded if any of the following conditions are satisfied:

1. Have other severe pain not related to the target disease that could cause confusion during pain assessment at screening or randomization.
2. Use prohibited concomitant therapies within 7 days prior to screening, or change to concomitant medications with restricted usage within 14 days prior to screening.
3. Have a clinically significant unstable nervous system, ophthalmological disease, hepatobiliary disease, respiratory disease, blood disease, cardiovascular disease, or mental disease within 12 months before screening.
4. Used EA treatment at the affected area within 1 month before screening.
5. Are pregnant, breastfeeding, or expecting a pregnancy during the study period.
6. Have implanted medical devices such as spinal cord stimulators, implantable drug delivery systems, pacemakers, automatic defibrillators, aneurysm clips, vena cava clips, and skull plates.
7. Have brain damage, symptomatic brain metastases, or epilepsy.
8. Have abnormal skin conditions that would prevent proper application of the EA treatment.
9. Are participating in other interventional clinical trials within 30 days before screening or present.
10. Have severe bleeding tendency: low doses of oral aspirin and a general dose of antiplatelet/anticoagulant medication generally do not limit study participation, but only when apparent risk is predicted by the investigators judgment.
11. Use immunosuppressive drugs.

### 2.4 Randomization and allocation concealment

The randomization table was generated using SAS Version 9.2 (SAS institute Inc., Cary, NC) to assign patients to the AC group or CA group in a 1:1 ratio by the block randomization method. After obtaining informed consent, participants who satisfy the inclusion/exclusion criteria will be allocated on a web-based basis according to this randomization table. A random allocation table will be created at the Medical Research Collaborating Center (MRCC) of SNUH, and will be operated through web random allocation. The outcome assessors, data managers, and statisticians will be blinded to the allocation.

### 2.5 Intervention

The EA treatment was modified from our pilot study,^[15]^ which was developed through a consensus of acupuncture specialists based on western medical acupuncture techniques as well as traditional meridian theory. EA treatment will be performed by Korean medicine doctors who graduated after 6 years of Korean medical college education and have at least 8 years of clinical experience. A total of 12 acupuncture sessions for 6 weeks will be performed using 0.25 × 40 mm disposable sterile acupuncture needles (Dongbang Acupuncture Inc., Chungnam, South Korea), which will be retained for 15 minutes. Up to 20 acupuncture points will be chosen by the practitioner based on traditional Asian medicine and western medical perspectives^[8,17]^ for local, segmental, and general stimulation. Local stimulation is defined insertion of the needle at a classical acupuncture point, tender point, or trigger point near the painful area. Segmental stimulation is insertion of the needle around area innervated by the same meridian or spinal nerve as that of the painful region. If direct needling to the local and segmental points exacerbates the pain, perisegmental points that are close to the affected segment, such as those above and below the area, or those on the opposite side of the equivalent contralateral segment will be used. Distal points which are located in the extremities for general stimulation will be chosen to treat the comorbidities of chronic pain according to traditional theory, such as insomnia (PC6, HT7, BL62, or KI6), depression/anxiety (PC6, HT7, LI4, or SP6), or fatigue (ST36, SP6, LU8, or GB20).

Electrical stimulation will be delivered using an EA device (ES-160, Ito Co., Ltd. Tokyo, Japan) with 2 Hz (local points), 2 to 6 Hz (local points or distal points), 120 Hz (segmental points), or 2 to 120 Hz (distal points) (altering every 2 seconds, 0.5 to 10 mA) approximately 80% of the maximum intensity.

### 2.6 Permitted and prohibited concomitant treatments

Existing oral and transdermal drugs (opioids, tramadol, non-steroidal anti-inflammatory drugs, steroid, muscle relaxants, topical capsaicin, neurotropin, prostaglandin, local anesthetics, vitamin B1 and B12, and nefopam) should be taken at the existing dose from screening to the end of the study. Depending on the pain intensity, additional prescription of oral acetaminophen is allowed as a rescue medicine for analgesic purposes. If acetaminophen is used, the participant should record the dose of acetaminophen and date in the patient diary. Anticonvulsants and antidepressants, which were being used to control symptoms of existing neuropathic pain, are also taken at the maintenance dose during the study period. Only when there are safety problems with the use of these drugs, dose reduction or discontinuation of these drugs will be permitted if necessary.

Subjects who received interventional therapies such as nerve block, iontophoresis, laser therapy, spinal cord irritation, and transcutaneous electrical nerve stimulation have a minimum washout period of 7 days prior to their first visit. These therapies are prohibited in the study period. Other forms of pain relief therapy for neuralgia such as psychotherapy and physiotherapy can be continued from 14 days prior to the first visit if the frequency of therapy does not change; however, these are not allowed to start during the study period.

### 2.7 Outcomes

#### 2.7.1 Primary outcome measurement

The proportion of participants who achieved improvement, as measured by the predefined composite efficacy outcome (CEO) [NRS decreased from baseline + pain interference scale (PIS) in the Brief Pain Inventory-Short Form (BPI-SF) decreased by more than 20% from baseline + “minimally improved”, “much improved”, or “very much improved” in patient global impression change (PGIC)] at 6 weeks after randomization, will be used as the primary outcome measurement.

#### 2.7.2 Secondary outcome measurements

Secondary outcomes include the differences in NRS score, the short-form McGill Pain Questionnaire, the BPI-SF, the Fatigue Severity Scale, the Hospital Anxiety and Depression Scale, the Medical Outcomes Study Sleep Scale, PGIC, EQ-5D-3L, PD-Q, Patient Satisfaction Scale, and incremental cost-effective ratio at 6 and 15 weeks after randomization will be measured.

#### 2.7.3 Safety outcome measurements

Safety will be examined on every visit by measuring vital signs, performing physical examinations, and recording adverse events (AEs). For vital signs, the degree of change after the treatment period compared to before the treatment period will be summarized as mean, standard deviation (SD), median, minimum, and maximum value. The results of the physical examination will be summarized by the frequency and ratio. AEs will be summarized by the number and ratio of patients who experience AEs and serious AEs. The human chorionic gonadotropin urine test will only be performed in fertile women at the first and fifth visits.

### 2.8 Sample size

This study was developed according to the following hypotheses:

H_0_: *p*_1_ = *p*_2_
H_1_: *p*_1_ ≠ *p*_2_

*p*_1_ = proportion of participants in the acupuncture group who achieved improvement, as measured by the predefined CEO after 6 weeks from baseline.

*p*_2_ = proportion of participants in the control group who achieved improvement, as measured by the predefined CEO after 6 weeks from baseline.

Based on our pilot study,^[15]^ the patients who achieved significant improvement was 44%, defined as a decrease in NRS, a reduction in PIS of BPI-SF scores of more than 20% from baseline, and change to “minimally improved”, “much improved”, or “very much improved” status, as measured by PGIC. Though there was no control group in the pilot study, we assumed that the conservative estimated proportion in the placebo group will be 10%.^[18]^ Assuming that the period and carryover effect does not exist, the difference in proportions of the 2 groups is 0.34 and within-subject SD is 0.34. Sixteen participants are required for each sequence group with a two-sided significance level of 5% (α = 0.05) and 90% power (1-β = 0.9). Consequently, a total of 40 patients (20 in each group) will be included, accounting for a potential 20% drop-out rate.

### 2.9 Statistical analysis

The analysis set will consist of a modified intention-to-treatment (ITT) analysis set, a per protocol (PP) set, and a safety set. The modified ITT set will include the participants who were randomized, received acupuncture or conventional treatments, and have at least 1 effect assessment after treatment. The PP set will include only participants who received at least 1 assessment and completed the study protocol without major deviation. The safety set will include any participants who received at least 1 acupuncture or conventional treatment after randomization. The modified ITT analysis will be the main analysis of this study.

For the descriptive analysis, Student *t* test or a Wilcoxon rank sum test will be performed for continuous data, and a Chi-Squared test or Fisher exact test will be performed for categorical data.

For a confirmatory analysis, the primary endpoint is the proportion of participants who show improvement, as measured by the predefined CEO at 6 weeks after randomization. The carry-over effect will be checked with a linear mixed model, and if the carry-over effect is not significant, the improvement frequency and ratio at 6 weeks for each treatment period in each sequence group will be summarized. The difference of the overall improvement rate (δ) between the groups will be estimated, and the null hypothesis will be rejected as follows:

$$\left|\frac{\overset{\circ }{\delta }}{{\overset{\circ }{\sigma }}_{d}/\sqrt{2n}}\right|>z_{0.05/2}$$

*σ*_*d*_: variance of the difference of the improvement rate between the groups within the subject

n: number of participants in each group

If the carry-over effect is significant, only data from treatment period A will be used and tested using the Chi-Squared test or Fisher exact test. Additionally, the carry-over effect, period effect, and sequence effect will be corrected using a generalized linear mixed model, and then the additional effects of acupuncture will be examined. At this time, the random effect, sequence, treatment period, and acupuncture treatment will be considered as fixed effects, and carry-over effect and period effect will be tested in terms of sequence order and treatment period, respectively. The term with a level of significance greater than 0.3 will be excluded from the model and the add-on effect of acupuncture treatment will be identified.

For secondary outcome measurements, continuous data will be summarized as mean, SD, median, and minimum/maximum value for each treatment period in each group, and compared between the 2 groups using a linear mixed model. Categorical data will be summarized as frequency and ratio for each treatment period in each group, and compared between the 2 groups using a generalized linear mixed model.

Statistical analyses will be performed by a statistician in the MRCC blinded to the group allocation using SAS Version 9.2 (SAS institute Inc., Cary, NC).

### 2.10 Data monitoring

The independent researcher will conduct regular monitoring to ensure quality control of the data. The researcher will monitor the written informed consent forms, compliance of protocol, and data documents during the study period. Monitoring also check for AEs.

### 2.11 Adverse events

Participants will be asked to report any AEs including those induced by EA throughout the study period at each visit. The expected AEs related with EA treatment include treatment-related pain, bruising, bleeding, or infection at the local site, nausea, dizziness, or anxiety.^[8,19]^

If AEs occur during the clinical trial, researchers will investigate the severity of symptoms, relevance to the intervention, action related to the intervention, treatments for AEs, and the result and seriousness of AEs. In the event of serious complications, researchers will provide adequate treatment to patients and terminate the EA treatment early if the patients find it difficult to continue the study. After that, a report will be prepared and submitted to the institutional review board (IRB).

### 2.12 Ethics and dissemination

The study was designed in accordance with the Helsinki Declaration to protect the participants and was approved by the IRB of the SNUH (H-1810-075-979) and the KHUKMH (KOMCIRB 2018-12-002). Only participants who voluntarily provide written consent after receiving a full description of the study prior to screening will be included. Participants are informed of the potential benefits, risks, alternatives, and responsibilities during the study by the researcher throughout the consent process. The results will be disseminated through a peer-reviewed journal and conference presentations.

## 3 Discussion

This is the study protocol for a prospective, open-labeled, randomized, cross-over clinical trial for patients with intractable neuropathic pain. This study aims to evaluate the effectiveness and safety of EA combined with conventional treatment compared to conventional treatment alone after 6 weeks of treatment.

This confirmatory trial was designed after adapting the results of our previous pilot trial^[15]^ which showed that the protocol was feasible and acceptable for a large scale trial considering the low dropout and high compliance rate. The study identified the appropriate dose of EA treatment, which was 12 sessions, once or twice a week, for 8 weeks. EA twice a week during the first 4 weeks led to an almost 50% reduction in pain intensity from baseline; however, EA once a week for the following 4 weeks did not have a clinically meaningful analgesic effect. Therefore, we will administer a total of 12 sessions of EA treatment twice a week for 6 weeks.

In our pilot study,^[15]^ affective dimensions, overall quality of life, and pain intensity were also improved after EA treatment. Since accompanying symptoms such as insomnia, anxiety, and depression, which are major comorbidities of chronic pain can affect pain intensity,^[4,20]^ it is necessary to identify the degree to which the analgesic effect of EA treatment correlates with the effect of these symptoms. Therefore, we measured the effect of EA treatment on these comorbidities with validated scales.

Some studies have suggested that pain intensity alone could not assess the value of interventions and overall experience of patients with chronic pain, and the functional aspect and quality of life should also be comprehensively considered.^[21]^ Therefore, integrating the assessment of pain intensity and functional disability is recommended for a trial assessing chronic pain, although there are some methodological disadvantages.^[22]^ Based on the results of our previous study, we defined the CEO to include pain intensity, various aspects of pain, and global assessment of the patient, and adapted it for this confirmatory trial.

To summarize, using data from our pilot study, this confirmatory trial was adapted with several improvements, and will assess the effectiveness and safety of EA in patients with refractory neuropathic pain.

## Acknowledgments

We would like to thank all members of the research team (doctors and clinical research coordinators) at KHUKMH and SNUH pain center.

## Author contributions

**Conceptualization:** Seunghoon Lee, Jee Youn Moon.

**Funding acquisition:** Seunghoon Lee, Jee Youn Moon.

**Methodology and project administration:** Seunghoon Lee, Jee Youn Moon, Sang Hoon Lee.

**Writing – original draft:** Seunghoon Lee, Suji Lee.

**Writing – review & editing:** Jee Youn Moon, Chang-Soon Lee, Yongjae Yoo, Sang Hoon Lee.
